# Variability of Vestibular Function in Pediatric Patients with Auditory Neuropathy Spectrum Disorder

**DOI:** 10.3390/audiolres16030089

**Published:** 2026-06-10

**Authors:** Guangwei Zhou, Kaitlyn Butler

**Affiliations:** 1 Department of Otolaryngology and Communication Enhancement, Boston Children’s Hospital, Boston, MA 02115, USA; kaitlyn.butler@childrens.harvard.edu; 2 Department of Otolaryngology, Harvard Medical School, Boston, MA 02115, USA

**Keywords:** Auditory Neuropathy Spectrum Disorder (ANSD), vestibular function, Vestibulo-Ocular Reflex (VOR), Vestibular Evoked Myogenic Potential (VEMP), pediatric vestibular testing

## Abstract

**Background/Objectives:** Auditory neuropathy spectrum disorder (ANSD) is a well-established hearing disorder in the pediatric population and is estimated to account for at least 10% of children with sensorineural hearing loss. Compared to auditory function, vestibular function in children with ANSD has not been well described in the past. The purpose of this study is to examine vestibular testing results in children with ANSD and to better characterize vestibular dysfunction in these children. **Methods:** A retrospective review of vestibular laboratory testing results was conducted in pediatric patients diagnosed with ANSD. Vestibular evaluation included vestibular evoked myogenic potential (VEMP), rotary chair test, video head impulse test (vHIT), and videonystagmography (VNG). **Results:** A total of 30 pediatric patients with ANSD were identified, including 18 boys and 12 girls, with a mean age of 4.5 years. Bilateral ANSD was found in 24 cases, while 6 cases were unilateral. Etiologies of ANSD included a history of hyperbilirubinemia in infancy, cochlear nerve dysplasia, genetic-related conditions, etc. Vestibular dysfunction was found in 12 cases, as indicated by at least one abnormal outcome in VEMP, vHIT, or rotary chair testing. Nineteen children were cochlear implant candidates and eventually underwent unilateral or bilateral implantation. **Conclusions:** Vestibular dysfunction is significant in pediatric patients with ANSD, and vestibular outcomes appear to be related to underlying etiologies. Formal vestibular evaluation is necessary to identify such vestibular losses, and these findings will be helpful to guide clinical management and rehabilitation strategies for these children.

## 1. Introduction

Auditory neuropathy spectrum disorder (ANSD) is a distinctive form of hearing impairment in which the neural conduction along the afferent auditory pathway is affected, while the outer hair cells in the cochlea are generally functional [1]. Pathologies associated with ANSD are varied, but certain auditory features are common, i.e., preservation of cochlear/outer hair cell function, such as the presence of otoacoustic emissions (OAEs) and/or cochlear microphonics (CM), alongside a lack of synchronized activity of the auditory nerve/afferents, as in auditory brainstem responses (ABRs) [2,3]. ANSD is well-established in the pediatric population, with studies reporting that it accounts for at least 10% of children with sensorineural hearing loss [4,5]. Hearing impairment in young children with ANSD can be identified early on. Whether pre-lingual or post-lingual, these children can be fitted with amplification to support speech and language development. If acoustic amplification is not successful, cochlear implantation may be considered an option to restore functional hearing [6,7].

Due to the intrinsic link between the auditory and the vestibular systems, it is not surprising that vestibular dysfunction may be present in children with ANSD. However, vestibular function has not been studied as extensively as auditory function in the pediatric population. Among published studies, there are conflicting reports regarding vestibular function in patients with ANSD. For example, abnormal caloric responses were reported in the majority of patients with ANSD in studies by Fujikawa & Starr, Hu et al., and Sujeet et al. [8,9,10]. In contrast, a study published by Akdogan et al. reported normal caloric responses in all three patients with ANSD [11]. Also, variable outcomes of vestibular myogenic potential (VEMP) have been reported in patients with ANSD [12,13,14]. Although the prevalence of vestibular dysfunction in pediatric patients with ANSD has been described [15,16], limited research has investigated the relationship between vestibular function and the underlying etiologies of ANSD.

This retrospective study aimed to investigate the occurrence of vestibular dysfunction in pediatric patients with ANSD and to explore the relationship between vestibular function and medical co-morbidities in these children. This study is also intended to provide important information for better clinical care and management of children with ANSD.

## 2. Materials and Methods

### 2.1. Patients

We conducted a retrospective review of vestibular laboratory testing results in a cohort of pediatric patients with a diagnosis of ANSD. The diagnosis of ANSD was based on clinical findings, including the results of hearing assessment (profound hearing loss), OAEs, and CM from ABR testing (presence of CM but no indefinable Wave I in ABR). These children were identified from our internal pediatric vestibular clinic database (from June 2016 to February 2026). Many of these patients were cochlear implant candidates and referred to us for pre-cochlear implant vestibular evaluation. Other patients were evaluated because of developmental/motor delays. Patient demographic data, auditory and vestibular outcomes, medical findings (including imaging and genetic results when available), and relevant medical history were collected from electronic medical records. We excluded the patients who had cochlear implant(s) prior to vestibular testing or insufficient information as described above. This study was approved by the Institutional Review Board (IRB number P00005505) at our hospital, and no parental consent is needed according to our institute policy.

### 2.2. Procedures

All patients underwent videonystagmography (VNG) and rotary chair testing using the Micromedical System 2000 (Micromedical Technologies by Interacoustics, Eden Prairie, MN, USA), along with cervical vestibular evoked myogenic potential (VEMP) testing using the Bio-logic Navigator Pro system (Natus Medical Inc., Schaumburg, IL, USA). In some children (e.g., ≥3 years), ocular VEMP was conducted, also using the Bio-logic Navigator Pro system. Additionally, video head impulse testing (vHIT) was conducted in children ≥ 3 years, using the ICS Impulse system by Natus. For a detailed description of vestibular testing techniques, please refer to previous publications [17,18]. All vestibular testing procedures were performed by a licensed audiologist with the aid of a trained assistant.

### 2.3. Data Analysis

A binary classification (normal vs. abnormal) was used in this study to categorize vestibular testing outcomes. If rotary chair testing was conducted using video goggles, results were considered abnormal if the vestibulo-ocular reflex (VOR) gain and/or phase were out of our established age-specific norms. If the rotary chair test was conducted using the observational camera, results would be considered abnormal if the VOR responses were absent or reduced. Cervical and ocular VEMP outcomes were classified as abnormal if responses were absent in the tested ear or the response threshold was higher than 90 dB. The vHIT was classified as abnormal if the VOR gain was below 0.7 and corrective saccades were observed in at least one semicircular canal. For each patient, vestibular function was classified as abnormal if one or more of the three tests, rotary chair, VEMP and vHIT yielded abnormal outcomes. Patients with known medical conditions associated with ANSD were categorized, and potential relationships with vestibular function were explored. Due to a relatively small number of subjects in this study, statistical analysis such as a chi-square test or any regression analysis would be invalid. Therefore, we chose to use a descriptive approach to describe our findings.

## 3. Results

### 3.1. Patients’ Demographics

A total of 30 pediatric patients were included in this study; 12 were female and 18 were male. Ages ranged from 7 months to 16 years, with a mean age of 4.5 years. Bilateral hearing loss/ANSD was present in 24 cases (80%), while unilateral hearing loss/ANSD was present in 6 cases (20%). Among these patients, 19 children were considered cochlear implant candidates. The majority of these children had implant surgery at the time of this retrospective study. A breakdown of patients’ demographics is shown in Table 1.

### 3.2. Vestibular Loss

Overall, evidence of peripheral vestibular loss (abnormal VEMP, vHIT or Rotary chair tests) was found in 12 out of 30 cases (40%) of children with ANSD. Specifically, abnormal rates were 40% (12 out of 30 cases) for the rotary chair test, 50% (5 out of 10 cases) for vHIT, 33% (10 out of 30 cases) for cervical VEMP, 50% (5 out of 10 cases) for ocular VEMP, and 38% (11 out of 29 cases) for the VNG test. A summary of these findings is presented in Table 2. Among the 12 patients with abnormal vestibular testing outcomes, dysfunction of both semicircular canals and otolith organs was found in 10 cases. Only two patients had abnormal VOR with intact VEMP responses. Although abnormal VNG findings were observed in 11 cases, only one case had central vestibular involvement, while the other 10 had minor ocular-motor anomalies (e.g., delayed latency in random saccade test).

### 3.3. Medical Conditions Associated with ANSD

After reviewing the medical records of 30 pediatric patients with hearing loss/ANSD, several associated medical conditions or morbidities were identified. The history of hyperbilirubinemia in the neonatal period was present in eight children. Non-syndromic conditions with genetic causes were found in five children, three of whom had otoferlin mutations. Syndromic conditions with a genetic cause were identified in four children; two had Perrault syndrome (one with *LARS2* gene mutation and one with *HAR2* gene mutation) and two had Charcot–Marie–Tooth disease (CMT) with *PMP22* gene mutations. Imaging studies revealed cochlear nerve dysplasia (absence or hypoplasia of the nerve) in three children. Also, two children with ANSD were found with VATER syndrome (VACTERL association). Nonetheless, probable causes could not be determined in seven cases with ANSD; see Table 3 for details. Among the 12 children with abnormal vestibular outcomes, five cases were found to have genetic causes (syndromic and non-syndromic), four cases had hyperbilirubinemia in the neonatal period, one case had VACTERL association, and two cases had unknown etiology. In contrast, no indication of vestibular dysfunction was found in the three cases with otoferlin mutations or in the three cases with cochlear nerve dysplasia.

## 4. Discussion

The prospect of vestibular dysfunction (e.g., abnormal caloric responses and gait disturbances) in children with ANSD was raised as early as in 2000 by Fujikawa and Starr [8]. Over the past two decades, vestibular studies in children with ANSD have been limited in volume, scale, and testing procedures [9,11,12,15,19]. Although pediatric vestibular assessment can be challenging and may not be widely available, progress has been made in recent years, and pediatric laboratory vestibular testing is more feasible than ever. Nonetheless, variability in reported outcomes across existing publications may present challenges for the clinical management of children with ANSD. Furthermore, clinical research investigating the relationship between vestibular function and underlying etiologies of ANSD in the pediatric population is scarce. Our current study was aimed at addressing these clinically relevant questions.

Overall, our study found evidence of vestibular loss in 40% of children with ANSD, which is comparable to the 42% reported by Nash et al. [15]. Moreover, loss of function in both semicircular canals (abnormal rotary chair test and/or vHIT) and otolith organs (abnormal cervical and/or ocular VEMP) were found in the majority of children with ANSD, 10 out of 12 cases (83%). Regarding abnormal VNG findings, only one case in which central involvement (cerebellum and/or brainstem) was suspected based on medical history and imaging results. The remaining 10 cases of ocular-motor anomalies observed during VNG testing may be attributed to maturation and attention factors. For example, naturally delayed maturation of the ocular-motor pathway among younger patients may not necessarily be pathological. In addition, some younger patients experienced greater difficulty maintaining attention during VNG testing. Nonetheless, our finding of vestibular dysfunction in a significant portion of young children with ANSD has important clinical implications. Healthcare professionals often focus on hearing loss in children with ANSD, while vestibular function may be overlooked. Since vestibular dysfunction may affect the development of motor abilities/skills and contribute to balance disturbances in young children, clinicians should be aware of clinical signs of vestibular dysfunction, such as delays in independent sitting, walking, and balance issues in children with ANSD. Considering that many of these children with ANSD may undergo cochlear implantation during their lifetime, it is beneficial to establish a baseline vestibular function in this population. This approach would provide a clearer understanding of the child’s medical status in determining proper management.

In our current study, we particularly paid attention to patients’ medical conditions related to ANSD. Historically, ANSD has been linked to a number of medical co-morbidities, such as neonatal hyperbilirubinemia and cochlear nerve dysplasia [2,3,5,20,21,22]. Not surprisingly, these two factors were found in our patients with ANSD as well, i.e., hyperbilirubinemia (*n* = 8) or cochlear nerve dysplasia (*n* = 3), and none had both, which was about 37% (11/30) of the cohort in the current study. All three children with ANSD associated with cochlear nerve dysplasia demonstrated normal vestibular function, indicating that the vestibular system was not involved in these cases. Our finding of 50% (4/8) of children with ANSD linked to hyperbilirubinemia having vestibular loss suggests that hyperbilirubinemia may affect the vestibular system, in addition to the auditory system. In recent years, genetic factors have also been implicated in ANSD [23,24,25]. In our study group, five out of nine, about 56%, children with ANSD associated with genetic mutation(s) were found to have vestibular loss. While vestibular impairment in patients with CMT was reported by Poretti et al. in 2013, their patients were all adults [26]. To our knowledge, vestibular findings in pediatric ANSD with CMT have been rarely described, and vestibular dysfunction in children with Perrault syndrome (carrying either *LARS2* or *HAR2* gene mutations) has not been reported previously; therefore, these observations may warrant further study. Another interesting finding in our study is that there was no vestibular loss in all three children with ANSD related to otoferlin mutations, and we were not able to find a similar study for comparison. In fact, the effects of otoferlin mutations on vestibular function have only been reported in animal studies [27].

The variability in vestibular function we found in children with ANSD suggests that the underlying etiology of ANSD might be a contributing factor. Future studies with a larger group of children with ANSD may provide further insight into this matter. The current study is limited by its retrospective nature and potential inclusion bias; therefore, it has reduced power to establish a universally applicable prevalence of vestibular dysfunction in children with ANSD. Also, we could not conduct any formal statistical analysis due to the small number of patients in subgroups. Nonetheless, our study includes sufficient data on vestibular testing and medical co-morbidities compared to previously published studies in this population.

## 5. Conclusions

Our findings highlight the significant presence of vestibular dysfunction in young children with ANSD. Vestibular dysfunction in ANSD was observed across various categories of etiology in this descriptive cohort. Prospective studies with a larger number of subjects are needed to determine whether specific etiologies of ANSD are associated with vestibular functional status. These results emphasize the importance of increased awareness of vestibular loss in this population to support appropriate clinical interventions. Formal vestibular evaluation is highly recommended to identify possible vestibular dysfunction in young children with ANSD. In order to increase referrals of children with ANSD for vestibular evaluation, extra efforts should be made in coordination with managing audiologists and other medical specialists. Clinicians who provide care to children with ANSD should better document any signs of possible balance and vestibular dysfunction, so that prompt referral is justified. Earlier identification of vestibular dysfunction in children with ANSD may lead to necessary management such as vestibular therapy.

## Figures and Tables

**Table 1:** Demographics of patients with ANSD.

Group	Demographic Information		
	Number of Patients	Mean Age (Range)	Hearing Loss/ANSD
**Male**	18	4.3 years (9 months–15 years)	Unilateral: 2 cases Bilateral: 16 cases
**Female**	12	4.9 years (7 months–16 years)	Unilateral: 4 cases Bilateral: 8 cases

**Abbreviations**: ANSD = auditory neuropathy spectrum disorder.

**Table 2:** Summary of vestibular findings from 30 children with ANSD.

Vestibular Testing	Normal/Total Cases	Abnormal/Total Cases
**Tests for Semicircular Canals**		
*Rotary Chair*	18/30	12/30
*vHIT*	5/10	5/10
**Tests for Otolith Organs**		
*Cervical VEMP*	20/30	10/30
*Ocular VEMP*	5/10	5/10
*VNG*	18/29	11/29

**Abbreviations**: VEMP = vestibular evoked myogenic potential; vHIT = video head impulse test; VNG = videonystagmography.

**Table 3:** Medical conditions associated with ANSD in 30 pediatric cases.

Medical Conditions/Diagnoses	Number of Cases
**Hyperbilirubinemia in infancy** **Genetic causes**	8
Non-syndromic	5
Syndromic	4
**Cochlear nerve dysplasia**	3
**VATER syndrome (VACTERL Association)**	2
**Other**	1
**Undetermined/unknown**	7
**Total**	30

## Data Availability

No new data were created or analyzed in this study. Data sharing is not applicable to this article.
